# Enhancing Cardiopulmonary Resuscitation Quality Using a Smartwatch: Neural Network Approach for Algorithm Development and Validation

**DOI:** 10.2196/57469

**Published:** 2025-05-05

**Authors:** Gaurav Rao, David W Savage, Gabrielle Erickson, Nathan Kyryluk, Pawan Lingras, Vijay Mago

**Affiliations:** 1 Department of Mathematics and Computing Faculty of Science Saint Mary's University Halifax, NS Canada; 2 Emergency Medicine Faculty of Family and Emergency Medicine NOSM University Thunder Bay, ON Canada; 3 School of Health Policy and Management Faculty of Health York University Toronto, ON Canada

**Keywords:** CPR performance, CPR feedback, neural network, smart health, smartwatch, sudden cardiac arrest, mobile phone, cardiac arrest, cardiopulmonary resuscitation, compressions, monitoring, emergency, network model, wearables, chest compression, efficacy, data collection

## Abstract

**Background:**

Sudden cardiac arrest is a major cause of mortality, necessitating immediate and high-quality cardiopulmonary resuscitation (CPR) for improved survival rates. High-quality CPR is defined by chest compressions at a rate of 100-120 per minute and a depth of 50-60 mm. Monitoring and maintaining these parameters in real time during emergencies remain a challenge.

**Objective:**

This study introduces a neural network model designed to predict and assess CPR quality using accelerometer data from a smartwatch.

**Methods:**

The study involved 83 participants performing CPR on mannequins, with accelerometer data collected via smartwatches worn by the participants. These data were aligned with gold-standard data from the mannequins. The accelerometer-derived compression data were segmented into 5-second intervals for training the neural network models. A total of 1226 neural network models were developed, incorporating variations in hyperparameters and dataset configurations to optimize performance.

**Results:**

The optimal model demonstrated the capability to accurately predict the number of compressions and the average compression depth within a 5-second interval. The model achieved an accuracy of ±3.8 mm for compression depth and an average deviation of 0.8 compressions. The results indicated that the neural network model could accurately assess CPR quality metrics, surpassing other models discussed in the literature. The large and diverse dataset used in this study contributed to the robustness and reliability of the model.

**Conclusions:**

This study validates the efficacy of a neural network model in accurately predicting CPR metrics using smartwatch accelerometer data. The model outperforms previous methods and shows promise for real-time feedback during CPR. Future work involves deploying the model directly on smartwatches for real-time application, potentially improving sudden cardiac arrest survival rates through immediate and accurate feedback on CPR quality.

## Introduction

Sudden cardiac arrest (SCA) is identified as a principal cause of mortality in North America, particularly among young athletes [1-4]. It can affect individuals irrespective of their lifestyle or health status, leading to either irregular or nonexistent heart rhythms [5,6]. As a consequence, the flow of blood to major organs is halted, depriving them of essential oxygen, resulting in tissue damage and potentially culminating in organ failure. The probability of survival decreases by about 10% with each passing minute without intervention; therefore, immediate and effective treatment is crucial to enhance survival rates by minimizing damage to tissues and organs [7,8].

Efforts to deliver prompt care to SCA victims continue, with emergency medical services (EMS) prioritizing SCAs to expedite emergency responses [9,10]. Public data indicate that the target for EMS response time to an SCA event is within 8-10 minutes [5,11-14]. The initial treatment involves performing cardiopulmonary resuscitation (CPR) and using an automated external defibrillator (AED) to assist in heart pumping and maintain blood flow to the brain and other vital organs. In certain instances, an AED can also restore the heart’s electrical activity [6,15].

Organizations such as the American Heart Association (AHA) and the Red Cross play a pivotal role in providing CPR and AED training to the public, empowering bystanders to administer early care to SCA victims until EMS arrives. Studies indicate that bystander-administered CPR significantly improves survival rates compared with scenarios where CPR is not administered [16-18]. The quality of CPR is critical, as high-quality CPR is associated with increased chances of survival. The AHA defines high-quality CPR as having a compression depth of 5-6 cm and a rate of 100-120 compressions per minute, standards that are also supported by the European Resuscitation Council [19,20].

Training the general public in CPR administration has emerged as a key objective for numerous organizations aiming to improve survival rates for SCA patients [21,22]. If a significant portion of the populace receives training in CPR, SCA victims stand a higher chance of receiving necessary immediate care until EMS arrive, thereby enhancing their survival prospects. Public training programs instruct participants on identifying SCA victims and initiating high-quality CPR. In these sessions, participants engage in CPR practice on mannequins while trainers offer real-time feedback on their performance, ensuring proficiency [23-25]. With advancements in technology, CPR performance can now be quantified, allowing feedback to be grounded in these measurements. Technologies used in training encompass sensor-equipped mannequins and CPR feedback devices.

Mannequins equipped with sensors accurately assess compression depth and frequency, providing trainers with metrics to deliver precise feedback to trainees. Beyond training devices, the industry also offers devices that can be placed on the chest of a mannequin or patient. These devices are equipped with accelerometers and pressure sensors to accurately assess CPR performance and transmit the information to another device for providing real-time feedback. The Laerdal CPRMeter2 is an example of such a device [26].

For SCA incidents, emergency call operators use technologies like tele CPR and video CPR to assist individuals in achieving high-quality CPR [27-29]. Upon identifying an SCA situation, EMS is dispatched, and the caller is guided over the phone on performing CPR. Nevertheless, the operator cannot gauge the quality of compressions administered, providing only verbal instructions to help the caller maintain an appropriate pace, which leaves compression depth uncertain. Video CPR, as an advanced approach, transitions communication from audio to video, allowing the operator to observe the CPR performance and offer real-time feedback. This technology requires advancements in emergency response systems to support video calling capabilities and necessitates video capability on the caller’s part. A limitation is its dependency on 2 bystanders—one to execute CPR and another to capture it.

Researchers have delved into next-generation CPR feedback technologies, including virtual reality and augmented reality–based devices [30-33]. These devices employ integrated cameras to capture and analyze compression depth and rate, displaying real-time statistics on the device screen to aid users in enhancing their CPR performance [34,35]. However, the widespread adoption of such advanced technologies encounters obstacles: they are not always accessible to those who do use them.

Therefore, the challenge lies in identifying a device suitable for real-world emergencies that can precisely measure CPR performance and provide appropriate feedback without the necessity for specialized hardware. The literature identifies smartphones and smartwatches as 2 potential solutions [36-38]. Both types of devices are equipped with sensors capable of evaluating CPR performance. Song et al [39] introduced a mobile app that leverages accelerometer data from smartphones to assess compression quality and provide feedback via screen displays and audio cues. Nonetheless, this approach faces limitations; it necessitates attaching the smartphone to the user’s arm, lacks details on data cleaning and noise removal, and does not consider variations in device orientation [40]. Similar challenges are evident in other algorithms designed for mobile CPR apps [41-43], with many focusing solely on training scenarios rather than real emergencies.

Gruenerbl et al [44] proposed a smartwatch app capable of measuring CPR parameters and offering visual feedback. This app analyzes accelerometer data to evaluate compression quality, identifying each positive peak on the y-axis as a compression and calculating the differences in y-axis peaks to determine compression depth. However, the study does not provide detailed algorithmic and data-cleaning methodologies for replication and comparison.

Lu et al [45] also proposed a smartwatch app alongside an algorithm for evaluating compression metrics. They tested using a Resusci Anne QCPR training manikin (Laerdal) and an android ASUS ZenWatch 2 (model WI501Q; ASUSTeK Computer Inc). The developed polynomial model predicts compression depth and rate from smartwatch accelerometer data. Although data were collected, its limited variability—compression counts between 80-140 and depths of 4-7 cm—fails to cover the wider range expected in real-world scenarios, nor does it elaborate on data cleaning or handling irregularities.

Using smartwatch accelerometer data presents several challenges, such as sensor white noise, gravitational effects, hand movements, and shifts in the watch’s position due to a loose fit. Filtering such noise from sensor data remains a significant challenge [46]. Employing noisy datasets can result in substantial variations in output results over time. To tackle noise filtration, we propose a neural network model trained on noisy data to predict compression performance, as detailed in the Methods section.

## Methods

### Overview

This section presents the technology and equipment used during the data collection, cleaning, and processing phases. A total of 83 participants from Thunder Bay Regional Health Sciences Centre (TBRHSC) took part in the data collection by performing CPR in a controlled simulation setting, using an Apple Watch, Apple iPhone, and a Laerdal mannequin for data capture [26,47].

### Equipment and Hardware

Participants collected accelerometer data with an Apple Watch Series 7 while performing CPR, which was then stored on an Apple iPhone 14. These devices, chosen for their significant presence in the wearable segment, were equipped with specially developed apps for data collection and analysis [48,49]. This study differs from previous ones by focusing on the potential of the Apple ecosystem for health care apps, rather than on Android devices. The apps were designed for compatibility with Apple iPhone 10 and newer, as well as Apple Watch Series 3 and later models.

CPR performance practice occurred on the Laerdal SimMan 3G mannequin, which is outfitted with sensors that record and provide feedback on key compression quality metrics such as depth, rate, and recoil. This aids in improving CPR quality [50]. Laerdal mannequins were chosen, as in other studies, for their precise data capture capabilities [39,40,44].

Data analysis was performed using a laptop with an i7 Intel processor, 32 GB RAM, and a 1TB hard drive, using Microsoft Excel and Python for data cleaning, processing, model training, and evaluation. Final analyses were done on a cloud server (Amazon Web Services) equipped with an NVIDIA A10G GPU (24 GB GPU Memory), 32 GB RAM, and 8 vCPUs [51].

### Data Collection

A convenience sampling method was used in this study. A notice was sent around the hospital and health care providers including doctors, nurses, respiratory therapists, and paramedics were recruited to participate. Data for this study were collected from 83 individuals over a 3-month period. Staff members from TBRHSC were invited to participate in the research, with their CPR experience ranging from none to more than 30 instances. Initially, participants received a brief orientation on CPR techniques and the benchmarks for high-quality CPR performance.

The simulation laboratory at TBRHSC, equipped with a Laerdal SimMan 3G mannequin capable of measuring compression depth, rate, and recoil, served as the setting for data collection. Upon arriving at the laboratory, participants received an Apple Watch containing the necessary data collection application. The participants were given training on how to perform high-quality CPR. After training, the participants were asked to perform CPR for 2 minutes, during which the data would be recorded. During these 2 minutes, participants were not provided with any feedback on their performance. A limited number of participants (n=41) performed another round of 2 minutes of CPR after taking a short 10-minute break.

When participants were ready to perform CPR after training, they were positioned correctly beside the mannequin, placing their hands on its chest, and initiated CPR compressions upon commencing data collection via the Apple Watch and mannequin. This procedure was carried out for 2 minutes, after which participants were advised to keep their hands in place on the mannequin to mitigate the potential introduction of noise in the data caused by removing their hands. The protocol was approved by TBRHSC and is attached as a Multimedia Appendix 1.

**Figure 1:**
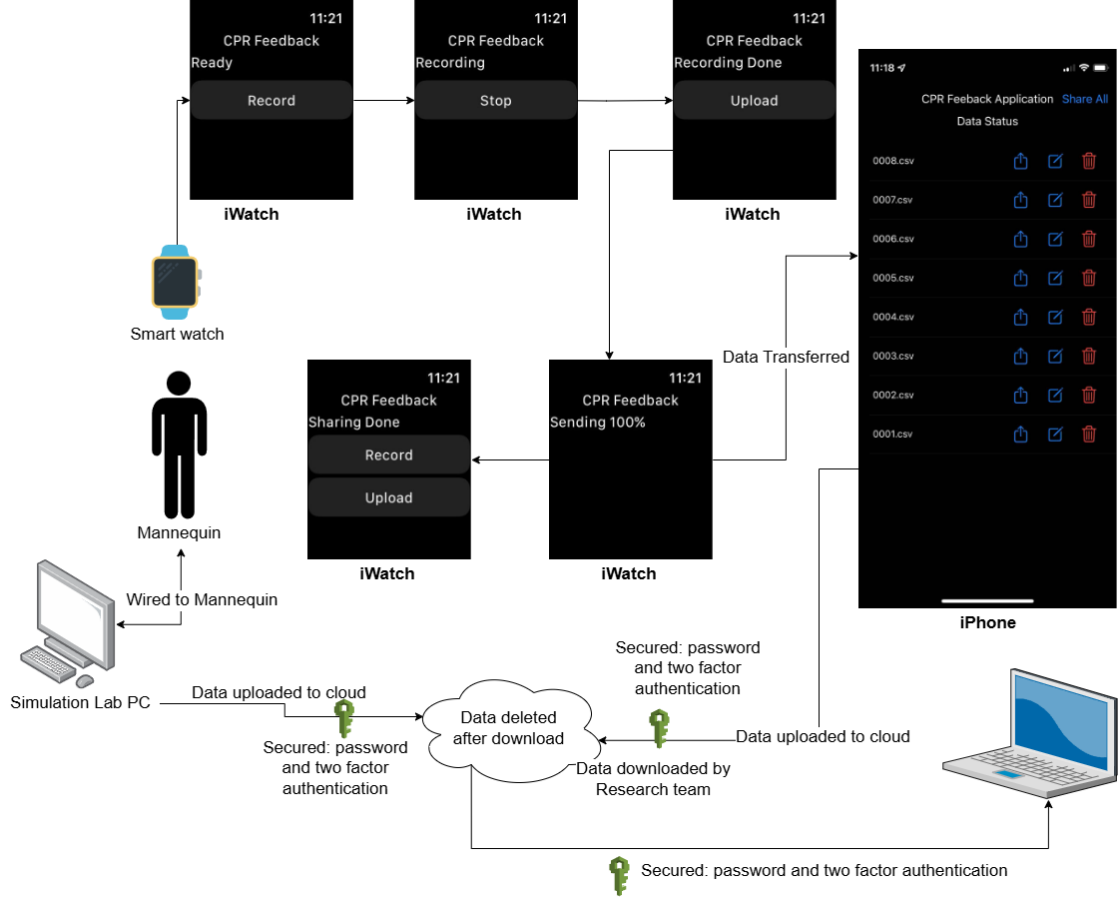
The flow diagram shows the screenshots of iPhone and Apple Watch apps, and the data flow used to collect the data during the study. CPR: cardiopulmonary resuscitation.

Subsequently, data from the Apple Watch and the mannequin were exported with a unique anonymous identifier to link the datasets without disclosing the identities of the participants. The process of data collection and the following data transmission is delineated in Figure 1. The data were securely transferred daily to the lead author’s workstation. The app on the Apple Watch recorded accelerometer data, including timestamps at a 50 Hz frequency. The mannequin’s data yielded detailed insights into each compression, providing metrics on depth, recoil completeness, start and end times of compression, and the peak time of compression. It is important to note that the mannequin’s time stamps were relative to the onset of the data collection period rather than an absolute start time.

In aggregate, the study compiled 256 minutes of CPR data, which included 27,844 compressions with an average depth of 44 (SD 4) mm. Of the 83 contributing participants, 27 were male, and 56 were female; 48 of these individuals had previously performed CPR on more than 10 occasions.

The sample size was determined by the number of eligible participants available during the data collection period, using a convenience sampling approach. While no formal power analysis was conducted, the sample size of 83 participants was considered sufficient to meet the study’s objectives. Notably, previous studies in similar domains have used smaller sample sizes—Gruenerbl et al [44] included 50 participants, and Lu et al [45] conducted their study with just 8 participants. In addition, some participants in our study performed CPR twice, resulting in 124 distinct datasets, further enhancing the dataset’s richness and utility for training and evaluation.

### Data Cleaning and Preparation for Model Training

#### Overview

**Figure 2:**
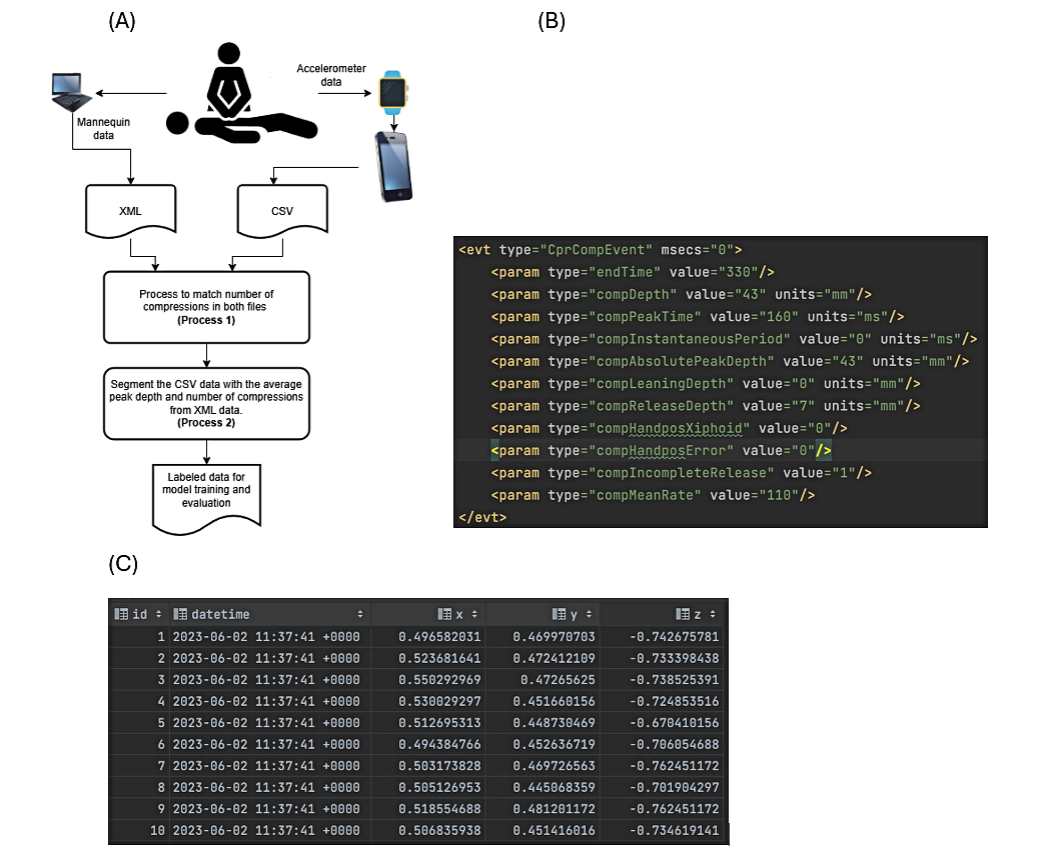
(A) The flow diagram illustrates the process for preparing the data for model training and sample data from XML and CSV files. (B) XML data file sample, showing data for 1 compression collected from the Laerdal mannequin. (C) CSV file sample, showing data collected from the Apple smartwatch. CSV: comma-separated value.

This study used data from 2 sources—an Apple Watch app and exported data from a mannequin. Each source’s data were recorded in separate files, but a unique identifier linked them for consistency throughout the data cleaning and preprocessing stages. Figure 2 illustrates the process of preparing the raw data for model training and the collected sample files.

The mannequin’s data, exported as a comprehensive list of compressions during each session, served as the gold standard due to the sensors’ precise compression depth, rate, and recoil measurements. Meanwhile, the Apple Watch provided time-stamped accelerometer data across the x-, y-, and z-axes, formatted as comma-separated values (CSVs). Both datasets included detailed compression information, enabling analysis of 2-minute CPR sessions.

For each session involving a pair of mannequin and smartwatch datasets, a 2-minute duration was recorded. The data were segmented into smaller portions to facilitate prompt feedback, potentially enabling real-time feedback. This segmentation process involved two critical steps: (1) aligning the datasets from the mannequin and the smartwatch to ensure their data corresponded accurately for analysis, detailed in subsection “Data Matching (Process 1)”; and (2) executing the chunking algorithm, detailed in the subsection “Data Chunking (Process 2).”

While real time user feedback was beyond this project’s scope, the data were examined to assess future applicability for this purpose. A 5-second analysis window was selected for potential feedback, where high-quality CPR—defined by approximately 10 compressions within this period—was used as the feedback standard. After receiving feedback, users would need time to comprehend and adjust their technique before the next feedback prompt.

#### Data Matching (Process 1)

The 2 data sources have different structures: the mannequin (XML file) records a sequence of compression events and their properties, while the smartwatch logs (CSV file) contain time-series accelerometer data. Because of this difference, synchronization cannot be based on time stamps. Instead, the datasets are aligned based on compression events, as the focus is on analyzing compression depth and frequency. The first step is to identify when compressions occurred using the accelerometer data, as explained further in this study. Once these events are detected, they can be matched with the corresponding compression depth and other properties from the XML file.

**Figure 3:**
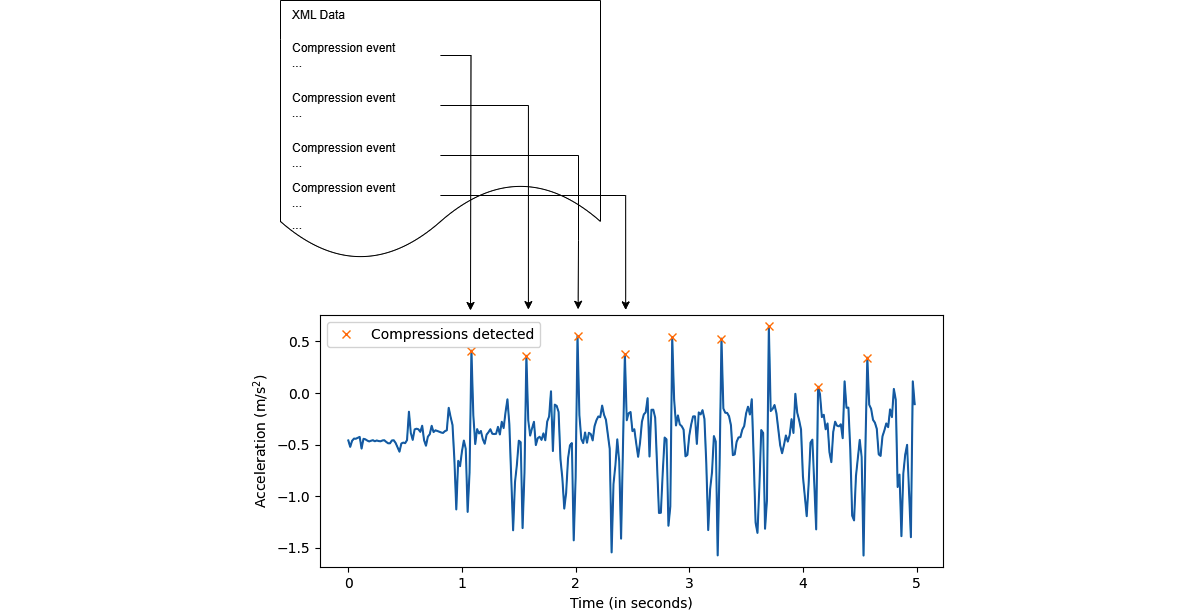
Process 1: The diagram illustrates a representative output showing the mapping of compressions in the XML data (mannequin data) with the compressions detected in the accelerometer data (smartwatch data).

A chest compression pattern typically indicates a peak in acceleration when the CPR performer compresses the chest, as shown in Figure 3. Due to sensor noise and the performer’s hand movement vibrations, various peaks can appear in the data; however, only a few of these should be considered valid compressions. The XML file from the mannequin specifies the number of compressions performed, which serves as the gold standard for creating an algorithm to identify compression peaks in the CSV file.

After analyzing the accelerometer data from the compressions, 3 variables were identified to help determine if a peak is a valid compression peak. First, the acceleration difference: the change in acceleration at the compression peak should be significant. Second, the window size is the time frame within which a peak can occur; for example, 2 compressions cannot happen within 5 microseconds. Third, the minimum acceleration peak helps filter out recoil peaks or peaks caused by vibrations or sensor noise. These values may vary from one compression to another, even if performed by the same person, due to factors such as user fatigue, incomplete recoil, changes in compression rate, or varying compression force.

**Figure 4:**
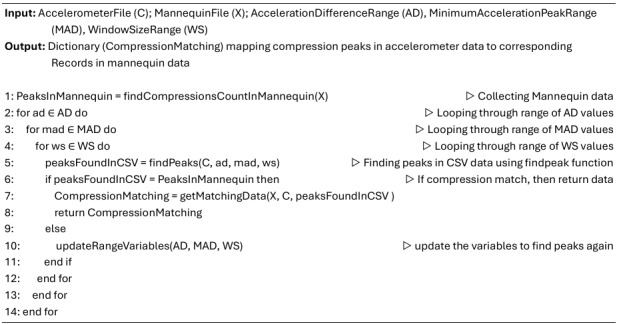
Pseudocode to generate matches between compressions in mannequin and accelerometer data files (algorithm 1).

To handle variability, algorithm 1 (Figure 4) was designed to match the “peaksFoundInCSV” with the peaks recorded in the XML file. The authors used SciPy’s *find_peaks* function to detect peaks in the CSV file based on 3 key variables—acceleration difference, window size, and minimum acceleration peak. Since these values varied across files, a script iterated through a range of values—acceleration difference (0.01-2), window size (1-40), and minimum acceleration peak (–1 to 2)—with step sizes of 0.2, 3.9, and 0.3, respectively, until the detected peaks matched those in the XML file. To verify accuracy, random manual checks were performed by generating graphs, similar to Figure 3, to ensure the peaks were correctly identified and aligned.

#### Data Chunking (Process 2)

**Figure 5:**
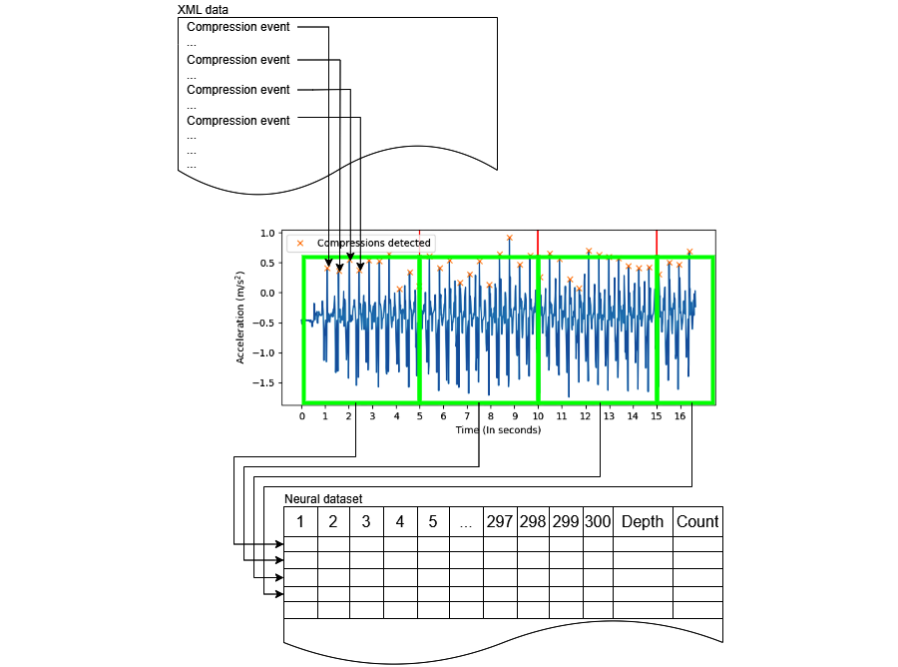
Process 2: Illustration of combining accelerometer and mannequin data to create the dataset for training the neural network model.

A 5-second interval was chosen as the optimal chunking duration, with the rationale for this selection explained in the previous section. Subsequently, the aligned datasets underwent segmentation into chunks for detailed analysis. Figure 5 illustrates the segmentation process applied to a representative dataset. Following the alignment of accelerometer data with the mannequin’s compression peaks, the accelerometer readings were divided into 300-point segments (indicated by a green box). Each segmented block (denoted by a green box) was then documented as a single record within the dataset used for training the proposed model, formatted in CSV. These records encompassed acceleration data for a 5-second interval (equivalent to 300 data points at a 60 Hz sampling rate), alongside the average compression depth and the total number of compressions recorded within that timeframe. For instances where the dataset was less than 5 seconds in length, the acceleration data was padded with zeros at the end to standardize it to a 5-second duration. The zero padding was performed on 124 records. The datasets prepared through this method were subsequently used to train the neural network model.

### Model to Predict CPR Performance

The aim of this study was to evaluate CPR performance by analyzing accelerometer data collected from smartwatches, which inherently included various forms of sensor noise, such as vibrations due to hand movements and accelerations not associated with chest compressions. The direct elimination of this noise was deemed impractical; therefore, the decision was made to use a supervised learning approach, specifically a neural network model, to tackle this intricate challenge.

**Figure 6:**
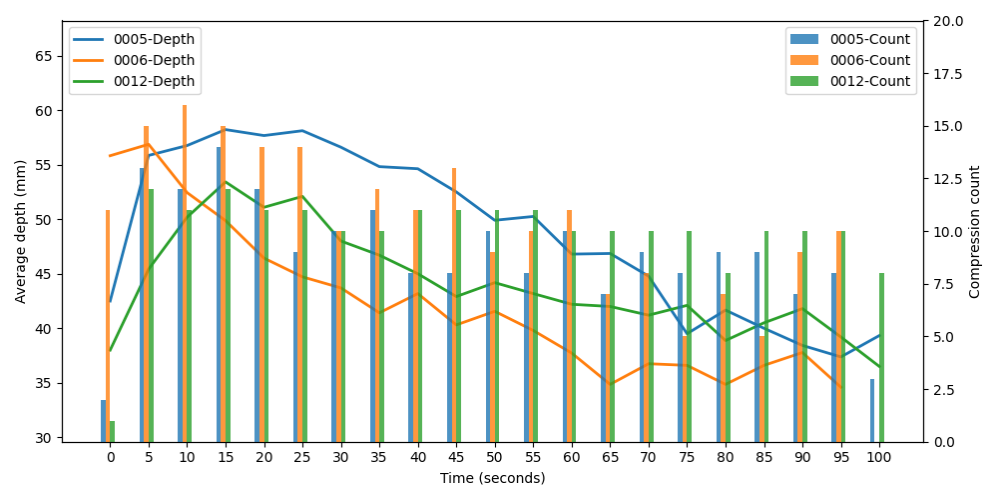
Variation in compression depth and compression rate among three participants over 100 seconds of CPR performance.

During model testing, the training data was randomly selected from 5-second chunks (2701 records), which could result in partial data from a participant appearing in both the training and test sets. However, since these chunks were not directly comparable, as determined during the review, the data were not split by participants. Although neural networks typically assume that data are independent and identically distributed, this study’s approach does not violate this assumption. Figure 6 illustrates the CPR performance of 3 participants over 100 seconds, summarized in 5-second intervals. Each chunk is treated as an independent data point, with no relationship to the previous or next chunk. The objective of this research is to randomly select a 5-second chunk and predict compression depth and count based on the accelerometer data, independent of past or future data. Since the model is trained to make predictions on isolated chunks rather than sequential patterns, the independent and identically distributed assumption holds, and data splitting by participants is not necessary. Figure 6 shows that compression depth declined over time and compression counts varied significantly. However, since the model does not rely on temporal dependencies, each chunk remains independent, justifying the decision not to split data by participants for training and testing.

The dataset was randomly split into 80% (2161/2701) training and 20% (540/2701) validation test sets. Each record consisted of 300 accelerometer data points, corresponding to a duration of 5 seconds at a 60 Hz sampling rate. The objective of the model is to predict both the number of compressions and their average depth during this interval.

The development of the model used the TensorFlow library within the Python programming environment [52]. To optimize the model’s performance, a combination of grid search and Hyperband techniques was used. In each experiment, grid search was used to explore a broad range of hyperparameters, identifying configurations that performed well or poorly. In addition, the Hyperband technique was applied to refine the search by excluding poorly performing hyperparameter sets, thereby narrowing the search space. This process was repeated iteratively, with grid search expanding the search range in each new experiment, followed by Hyperband reducing it to eliminate ineffective configurations. The key hyperparameters adjusted included the number of hidden layers, layer sizes, number of epochs, batch sizes, and dropout rates. The objective was to determine the most effective combination of hyperparameters that would allow the model to accurately interpret accelerometer data, despite sensor noise, and reliably assess CPR quality based on smartwatch data.

To evaluate the normality of the data and assess the correlation between actual and predicted values, 2 statistical tests were conducted. First, the Shapiro-Wilk test was performed on the expected depth and expected compression count to determine if the data followed a normal distribution. The results showed *P* values of 2.69×10⁻³⁶ and 2.80×10⁻⁴⁵, respectively, indicating that both datasets deviated significantly from normality. Given this, Spearman rank correlation was chosen to measure the relationship between expected versus predicted depth and compression counts since it does not assume normality.

### Ethical Considerations

The research ethics evaluation for this study was reviewed and approved by the TBRHSC Research Ethics Board (2022519), with all procedures conducted in accordance with the ethical standards of the responsible institutional committee and the principles outlined in the World Medical Association Declaration of Helsinki. Written informed consent was obtained from all participants before their inclusion in the study. To maintain privacy and confidentiality, all data collected were deidentified at the point of collection, with each participant assigned a unique identifier; a separate, secure document mapping participant identities to these identifiers was maintained independently and was not linked to the research data. Participants were compensated with a US $5 gift card for their participation.

## Results

Table 1 contains the demographic details of participants involved in the data collection process. A total of 83 individuals contributed, resulting in 124 mannequin-smartwatch datasets, with 41 participants completing 2 rounds of CPR. The sample included 56 females and 27 males, spanning various age groups, with the largest group being younger than 30 years old. Participants also varied in their previous CPR experience, ranging from no previous attempts to over 30, offering a diverse dataset in terms of skill level and background.

**Table 1:** Demographic details of participants in the data collection process.

Description			Number of participants, n
Number of mannequin-smartwatch datasets			124
Number of participants			83
Participants performing 2 rounds of CPR^a^			41
**Sex**			
	Female	56	
	Male	27	
**Age groups (y)**			
	<30	42	
	30-39	27	
	40-49	8	
	>50	6	
**Previous CPR attempts**			
	0	12	
	1-9	23	
	10-19	10	
	20-29	14	
	30+	24	

^a^CPR: cardiopulmonary resuscitation.

A series of experiments were systematically conducted, exploring various hyperparameters and datasets, until no notable improvements in outcomes were observed. This investigative process entailed 12 different experiments to find the optimal set of hyperparameters. Within each trial, data on the variations in hyperparameters were documented, noting the discrepancies between anticipated and actual compression depths and counts. Given that the variance between expected and observed results could manifest in negative figures, potentially skewing average calculations, absolute values were used for a more accurate determination of the average and median. These analytical steps informed adjustments in hyperparameters for ensuing experiments aimed at enhancing performance.

Throughout this extensive testing phase, a total of 1226 iterations were conducted, each exploring different hyperparameter settings. The specific settings and the best outcomes from each experiment are presented in Table 2, while detailed information on each experiment is provided in Multimedia Appendix 2. Summarizing the testing, the most effective model across all iterations was characterized by the following hyperparameters: hidden layers configured as (2000, 1000, 250, 100, 50), a batch size of 2000 epochs, and a dropout rate of 10% after every 2 layers. Predictions from this model demonstrated an average absolute loss in compression depth of 3.8 (SD 0.16) mm and in compression count of 0.8 (SD 0.05) counts, with a median absolute loss for compression depth at 2.59 (IQR 0.24) mm and for compression count at 0.03 counts. Throughout the course of the experiments, 27 models were identified as capable of predicting an average absolute compression depth below 4.0 mm.

The Spearman correlation coefficients were 0.80 and 0.78, respectively, with *P* values of 1e-3, indicating a strong and statistically significant positive correlation between actual and predicted values for both compression depth and compression count. These findings suggest that the model effectively predicts compression depth and count, even though the underlying data distribution is not normal.

**Table 2:** Summarizing hyperparameters for each experiment and the best results achieved.

Experiment	Epochs	Batch size	Layer sizes	Number of layers	Connection dropout	Best result (compression depth, count)
1	10	3, 5, 7, 9, 11	10, 35, 60, 85, 100, 400, 700, 1000, 1200	3	10% after every 2 layers	±4.8 mm, 1.2 counts
2	100, 500	6, 12, 18	10, 35, 60, 85, 100, 400, 700, 1000, 1200	3, 4	10% after every 2 layers	±4.0 mm, 0.9 counts
3	500, 750	18, 24	5, 40, 100, 500, 900, 1300	4, 6	10% after every 2 layers	±4.8 mm, 1.2 counts
4	500, 750	18, 24	5, 40, 100, 500, 900, 1300	4, 6	10% after every 2 layers	±3.9 mm, 0.9 counts
5	500, 750	18, 24	5, 10, 60, 100, 400, 700, 1000	4	10% after every 2 layers	±3.9 mm, 0.8 counts
6	500, 750	6, 24	5, 10, 50, 100, 500, 1000, 4000	4, 5	10% after every 2 layers	±6.2 mm, 1.2 counts
7	1000	3	5, 10, 50, 100, 500, 1000, 4000	5	10% after every 2 layers	±4.0 mm, 0.9 counts
8	1000	12	5, 10, 50, 100, 500, 1000, 4000	5	No dropouts	±3.9 mm, 0.9 counts
9	1000	3, 12	5, 10, 50, 100, 500, 1000, 4000	3	No dropouts	±4.1 mm, 0.9 counts
10	100, 500, 1000	3, 9	5, 10, 25, 50, 75, 100, 250, 500, 1000, 1250, 1500, 1750, 2000, 2250, 2500, 2750, 3000, 3250, 3500, 3750, 4000	3	10% after every 2 layers	±4.1 mm, 0.9 counts
11	100, 1000	3, 128	5, 10, 25, 50, 75, 100, 250, 500, 1000, 1250, 1500, 1750, 2000, 2250, 2500, 2750, 3000, 3250, 3500, 3750, 4000	5	10% after every 2 layers	±3.9 mm, 0.8 counts
12	1000, 2000	256, 1024	5, 10, 25, 50, 75, 100, 250, 500, 1000, 1250, 1500, 1750, 2000, 2250, 2500, 2750, 3000, 3250, 3500, 3750, 4000	5	10% after every 2 layers	±3.8 mm, 0.8 counts

## Discussion

### Overview

This study introduces a neural network model capable of using accelerometer data from a smartwatch to guide users in delivering high-quality CPR. To facilitate this, apps for both Apple Watch and iPhone were developed to gather accelerometer data during CPR. This research compiled data from 83 participants using Apple Watches to perform CPR on a mannequin, resulting in a total of 27,844 chest compressions. A total of 1226 experiments were conducted to evaluate different hyperparameter settings. The most effective model used hidden layers (2000,1000,250,100,50), a batch size of 2000, and a 10% dropout after every 2 layers. This model achieved an average absolute loss of 3.8 mm in compression depth and 0.8 in compression count, with median losses of 2.59 mm and 0.03 counts, respectively. In total, 27 models achieved an average absolute compression depth loss of less than 4.0 mm.

### Principal Results

Considering mannequin data as the benchmark, the neural network was trained with the accelerometer data to emulate the mannequin’s feedback. After undergoing 1226 iterations of model refinement, the optimal model emerged, capable of estimating compression depth with an average discrepancy of 3.8 mm and compression count with an average discrepancy of 0.8 counts across 5-second intervals. This investigation underscores the potential of a neural network model to surpass existing models in predicting CPR quality, underpinned by a more extensive and realistic dataset than those previously reported in the literature [40,44,45,53-55].

### Comparison With Previous Work

Numerous studies have highlighted significant enhancements in CPR quality when performers receive real-time feedback through intelligent technologies [40,53,56]. Gruenerbl et al [44] proposed a smartwatch app capable of measuring CPR parameters and offering visual feedback. This app analyzes accelerometer data to evaluate compression quality, providing user feedback to achieve high-quality compressions. Lu et al [45] also proposed a smartwatch app alongside an algorithm for evaluating compression metrics. However, these proposed solutions are developed on limited datasets and do not provide sufficient details to reproduce or enhance the system. Dedicated devices, such as the Laerdal CPRMeter2, which can be placed on the chest of a mannequin or patient, are available for purchase. These devices are equipped with accelerometers and pressure sensors to accurately assess CPR performance and provide real-time feedback [26]. However, these devices are expensive and must be carried by the user at all times, as emergencies can occur at any moment.

### Limitations

This study demonstrates that the accelerometer data from the smartwatch can be used to provide real-time feedback to the CPR performer and can help increase the survival chances of the patient. However, there are a few limitations worth noting. First, the developed model focuses on measuring compression depth and count but does not account for compression recoil. Compression recoil plays an important role in performing high-quality CPR, as it allows the patient’s chest to fully expand. This full expansion enables more air to enter the patient’s body. Compression recoil can also be measured using acceleration data from the smartwatch and compared with sensor data from the mannequin, similar to how compression events are measured. In future research, the authors plan to include this factor as well.

Second, the Apple Watch Series 7 was the exclusive device used to collect data during this study. However, sensors in smartwatches vary between brands, models, and versions, each having its own set of white noise that may impact the results. Therefore, it is necessary to investigate how other devices with different sensors perform with the presented model and whether the model needs to be tuned for each device type. In future studies, the authors plan to use an Android device to cover a broader spectrum of sensors.

Third, during data collection, it was observed that the sensor data frequency fluctuated between 48-60 Hz, although the collection rate was set to 60 Hz. This fluctuation could be due to several reasons: slower processing on the device, the device’s inability to write data at that frequency to the file, or the device’s processing speed being unable to read the data from the sensors at this speed. Therefore, it is important to investigate how the model’s performance would be affected if the data frequency were lowered.

Fourth, while working with the Apple Watch, the authors found a limitation that restricts its use during emergency events. The Apple Watch only allows an app to run in the background for 30 seconds before it is suspended, after which the app can only execute its tasks intermittently. This restriction hinders the use of the smartwatch for providing real-time feedback during emergencies.

Finally, in data segmentation, the acceleration data were chunked based on a fixed number of data points (300, representing 5 seconds of data at 60 Hz). This chunking may occur during a compression event, potentially splitting the compression event into 2 parts, which the algorithm may not recognize, resulting in missed compressions. In future research, the authors plan to address this problem by introducing an advanced algorithm that can recognize the completion of a compression event and then segment the data accordingly to avoid splitting compression data.

### Conclusions

This study presents a neural network model designed to gauge the rate and depth of CPR compressions accurately. The model has undergone training on a large dataset, ensuring a robust foundation for its predictive capabilities. Its accuracy is remarkably close to the measurements obtained from a CPR mannequin, serving as a benchmark for comparison. Notably, this model demonstrates superior performance when compared with other models discussed within the existing literature, establishing it as a significant advancement in the field of medical emergency training and response analysis. Implementation of this model in real-world scenarios could significantly improve SCA survival rates.

## Appendix

Multimedia Appendix 1 Informed consent form.

Multimedia Appendix 2 Experiment details.

## Abbreviations

AED: automated external defibrillator
AHA: American Heart Association
CPR: cardiopulmonary resuscitation
CSV: comma-separated value
EMS: emergency medical services
SCA: sudden cardiac arrest
TBRHSC: Thunder Bay Regional Health Sciences Centre

## Data Availability

The data supporting the findings of this study are available from the corresponding author upon reasonable request. Access to the data may be granted for research purposes, subject to ethical considerations and approval.
