# Supplementary material for: Enhancing Cardiopulmonary Resuscitation Quality Using a Smartwatch: Neural Network Approach for Algorithm Development and Validation
Source: JMIR Mhealth Uhealth. 2025 May 5;13:e57469. doi: 10.2196/57469 (PMC12089875; doi:10.2196/57469)
Supplement: Multimedia Appendix 2 [file mhealth_v13i1e57469_app2.docx]

**Experimental Details**

The initial trial set the hyperparameters as follows: epochs ranged from 10 to 1,000, batch sizes varied from 3 to 11, and layer sizes were set between 10 and 1,200 across three hidden layers, with a 10% reduction in connection dropout implemented after every two hidden layers. This structure established the input layer to accommodate 300 points, while the output layer was configured to include two points. The strategy for structuring hidden layers dictated that the central layer would achieve the maximum size, with sizes incrementally increasing from the input towards this midpoint before decreasing towards the output layer. An illustrative configuration for a three-layer model might progress from 300 to 1,000 at the peak, then taper down to 100 at the output layer (300-700-1000-100-2).

The first experiment involved 64 different model iterations, showcasing the impact of varying layer sizes, with the largest hidden layer reaching 1,000 and the epoch count set at 10. This particular iteration yielded the most accurate prediction for compression depth, with an average absolute deviation of 4.8mm and for compression count, an average absolute deviation of 1.2 counts. Notably, this experiment underscored the critical influence of layer size configurations on model outcomes, as identical batch sizes and epoch durations led to significantly diverse results based on the arrangement of the final hidden layer.

In the second experiment, variability was introduced in the hyperparameters batch size, epochs, and the number of layers, resulting in 36 distinct combinations of these variables. Among these iterations, the most successful outcomes were observed when the batch size and epoch count were set to 18 and 500 respectively, with the four hidden layers. This configuration yielded the most accurate prediction of compression depth, registering an average absolute deviation of 4.0mm, and a similar accuracy in compression count with an average absolute deviation of 0.9 counts.

For the third experiment, adjustments were made to increase epochs to [500, 750], batch sizes to [18, 24], and the number of hidden layers to [4, 6], while also reducing layer size options. A modification was applied to the layer combination approach, prioritizing the largest hidden layer initially and progressively diminishing layer sizes towards the output. This methodology facilitated 30 unique iterations exploring various configurations of hyperparameters and layer structures. The best iteration within this set achieved an average absolute loss of 3.9mm for compression depth and 0.9 counts for compression count, with the top three iterations all featuring four hidden layers, batch sizes of 24, and 750 epochs.

The fourth experiment maintained the hyperparameter settings of the third experiment but introduced a significant modification to the dataset used for training. Instead of solely relying on the raw accelerometer data, this trial added a smoothed version of the entire dataset, effectively doubling its size. The data was smoothed by applying a moving average filter with a window size of five. Despite this alteration in the dataset, the impact on the experiment’s outcomes was minimal, resulting in findings that closely paralleled those of the third experiment.

In the fifth experiment, attention was directed towards evaluating smaller hidden layer sizes. The approach for configuring layers reverted to positioning the central hidden layer as the largest, with preceding layers incrementally increasing towards this peak and subsequent layers diminishing towards the output. This adjustment led to 30 viable hyperparameter combinations. The most accurate iteration within this trial achieved an average absolute deviation in compression depth of 3.9mm and in compression count of 0.8 counts. Among the top-performing configurations, four hidden layers, 750 epochs, and batch sizes set at 24, and 18 were common. When comparing the leading outcomes from the fourth and fifth experiments, a slight improvement was noted in the latter.

The sixth experiment introduced alterations in hyperparameters, specifying batch sizes of 6 and 24, hidden layer counts of 4 and 5 and adding a layer size of 4,000. Unfortunately, this led to a decline in performance, with the optimal prediction exhibiting a substantial average absolute loss in compression depth of 6.2mm.

In the subsequent seventh experiment, epochs were elevated to 1,000, batch size was minimized to 3, and the model was structured with five layers. These modifications markedly enhanced the results, yielding the prediction of an average absolute compression depth of 4.0mm and an average absolute compression count of 0.9.

The eighth experiment saw further adjustments from the seventh, notably increasing the batch size to 12 and eliminating connection dropouts. While the best outcomes of the seventh and eighth experiments were closely matched, a comparative analysis revealed that all iterations from the eighth experiment consistently outperformed the third-highest result of the seventh, indicating a refined optimization of hyperparameters in the latter experiments.

For experiment nine, batch sizes were designated as [3,12], and the model was configured with three hidden layers. The outcomes from this trial were notably inferior to those observed in the eighth experiment. The most accurate iteration within this series estimated the compression depth with an average absolute deviation of 4.1mm and the compression count with an average absolute deviation of 0.9 counts.

The tenth experiment explored a broader range of hyperparameters, setting epochs at [100,500,1000], batch sizes at [3,9], and maintaining the number of layers at three. A 10% dropout rate was applied after every two layers, and layer sizes varied from 5 to 4,000, applying a strategy where layer sizes increased towards the midpoint before decreasing towards the output. This extensive exploration resulted in 519 potential hyperparameter combinations. The iteration yielding the most accurate results, the compression depth with an average absolute loss of 4.1mm and the compression count with an average absolute loss of 0.9 counts, indicating that the findings from this extensive trial did not surpass the performance metrics established in earlier experiments.

In the eleventh experiment, adjustments were made to three hyperparameters: epochs were set to [100,1000], batch size to [3,128], and layer size was fixed at [5]. The strategy for layer configuration aimed to establish the initial hidden layer as larger than the input layer, with each subsequent layer diminishing in size relative to its predecessor. This approach generated 107 potential model iterations. The optimal iteration within this series estimated the compression depth with an average absolute deviation of 3.9mm and the compression count with an average absolute deviation of 0.8 counts. Compared to the results from the tenth experiment, the eleventh experiment showcased superior performance, yielding ten models that outperformed the top model from experiment ten.

In the twelfth experiment, modifications were applied to two hyperparameters: epochs were adjusted to [1000,2000], and batch sizes were set to [256,1024]. This trial yielded slight enhancements in predicting both compression depth and count over its predecessor. The best iteration from this experiment achieved a compression depth prediction with an average absolute loss of 3.8mm and a compression count prediction with an average absolute loss of 0.8 counts.
