# Supplementary material for: Enhancing Cardiopulmonary Resuscitation Quality Using a Smartwatch: Neural Network Approach for Algorithm Development and Validation
Source: JMIR Mhealth Uhealth. 2025 May 5;13:e57469. doi: 10.2196/57469 (PMC12089875; doi:10.2196/57469)
Supplement: Multimedia Appendix 1 [file mhealth_v13i1e57469_app1.pdf]

Thunder Bay Regional
Health Sciences
Centre

980 rue Oliver Road
Thunder Bay ON
P7B 6V4 Canada

Tel: (807) 684-6000
www.tbrhsc.net

Research Ethics Board

November 22, 2022

Dr. David Savage dsavage@nosm.ca
TBRSHC, Emergency Department
980 Oliver Road
Thunder Bay, ON P7B 6V4

Re: **Project Number:** TBRHSC REB # 2022519
Project Title: Measuring CPR feedback using smartwatch technology
REB Expiry Date: 22-Nov-2023

Dear Dr. Savage,

Thank you for your submission to the Thunder Bay Regional Health Sciences Centre Research Ethics Board (TBRHSC REB). The above noted application has been reviewed and approved through the delegated review process for the TBRHSC REB. **TBRHSC REB approval is granted based on the following documentation:**

- REB Application v1.1 dated 15-Nov-2022
- Protocol v1.1 dated 15-Nov-2022
- Data Collection Points and Sources v1.0 dated 26-Jul-2022
- Informed Consent Form v1.0 dated 26-Jul-2022
- Study Poster v1.0 dated 25-Jul-2022
- Partiipant Survey v1.0 dated 20-Jul-2022
- Budget v1.0 dated 16-July-2022
- COI Forms – GR, VM

The delegated approval for this study will be reported to the full REB at their next meeting. TBRHSC REB is guided by the Tri-Council Policy Statement: Ethical Conduct for Research Involving Humans 2018 and ICH: Good Clinical Practice (GCP) guidelines.

In addition to this TBRHSC REB approval, all projects at TBRHSC require Research Program review. Prior to beginning your REB approved project, research teams must receive written notification for final project authorization from the Research Program.

Continuing ethics review is the responsibility of the Principal Investigator. During the course of your research, any serious adverse events, changes in the approved protocol, consent form or other information needs to be submitted for review to the REB using the appropriate forms. REB approval is required should your project extend beyond the approval period noted above. Upon completion/termination of the study you are required to submit a Study Completion Report. All forms are available at:

"https://tbrhsc-tbrhri.researchservicesoffice.com/Romeo.Researcher"

Yours Sincerely,

Bill Gregorash, BA, MA, PhD Management Date
Chair, TBRHSC REB

Research Ethics Board

The TBRHSC Research Ethics Board operates in compliance with and is constituted in accordance with the requirements of:

- TCPS 2 - 2018 Edition of the Tri-Council Policy Statement: Ethical conduct for Research Involving Humans,
- ICH Good Clinical Practice: Consolidated Guideline - ICH E6(R2)
- Part C division 5 of the *Food and Drug Regulations* of Health Canada dated 21-Aug-2019, and
- The provisions of the Personal Health Information Protection Act 2004 and its applicable regulations
- TBRHSC REB is registered with the US department of Health and Human services under IRB registration #00004396

Description of Research Team

Principal Investigator:	Dr. David Savage<br>TBRSHC, Emergency Department<br>980 Oliver Road<br>Thunder Bay, ON P7B 6V4<br>dsavage@nosm.ca
Qualified Investigator:	N/A
Co-Investigator(s):	Gaurav Rao<br>Saint Mary's University,<br>Department of Mathematics and Computer Science<br>Raog726@gmail.com<br><br>Vijay Mago<br>Lakehead University<br>Department of Computer Science<br>vmago@lakeheadu.ca
Best Contact for Project:	Shalyn Littlefield<br>TBRHRI, CRSD<br>Room 2163, 980 Oliver Road<br>Thunder Bay, ON P7B 6V4

Research Ethics & Authorization Office

Room 2167 – Level 2
980 Oliver Road
Thunder Bay, Ontario
Canada P7B 6V4

Tel.: (807) 684-6422
e-mail:
TBR_REO@tbh.net
